# Function does not follow form in gene regulatory circuits

**DOI:** 10.1038/srep13015

**Published:** 2015-08-20

**Authors:** Joshua L. Payne, Andreas Wagner

**Affiliations:** 1 University of Zurich, Institute of Evolutionary Biology and Environmental Studies, Zurich, Switzerland; 2 Swiss Institute of Bioinformatics, Lausanne, Switzerland; 3 The Santa Fe Institute, Santa Fe, New Mexico, United States of America

## Abstract

Gene regulatory circuits are to the cell what arithmetic logic units are to the chip: fundamental components of information processing that map an input onto an output. Gene regulatory circuits come in many different forms, distinct structural configurations that determine who regulates whom. Studies that have focused on the gene expression patterns (functions) of circuits with a given structure (form) have examined just a few structures or gene expression patterns. Here, we use a computational model to exhaustively characterize the gene expression patterns of nearly 17 million three-gene circuits in order to systematically explore the relationship between circuit form and function. Three main conclusions emerge. First, function does not follow form. A circuit of any one structure can have between twelve and nearly thirty thousand distinct gene expression patterns. Second, and conversely, form does not follow function. Most gene expression patterns can be realized by more than one circuit structure. And third, multifunctionality severely constrains circuit form. The number of circuit structures able to drive multiple gene expression patterns decreases rapidly with the number of these patterns. These results indicate that it is generally not possible to infer circuit function from circuit form, or vice versa.

Gene expression is tightly regulated in both space and time^1^,^2^. Much of this regulation is carried out by sequence-specific DNA-binding proteins known as transcription factors (TFs), which activate or repress the expression of genes by promoting or preventing the recruitment of RNA polymerase. TFs often regulate the expression of other TFs, resulting in the formation of small regulatory circuits that are nested within a cell’s larger regulatory network^3^. The gene expression patterns of such circuits embody crucial biological functions, ranging from chemotaxis in bacteria^4^ to limb development in vertebrates^5^.

In many biological systems, form or structure can hint at function. Examples range from the fusiform shapes of fast-swimming animals like sharks and squid to the three-dimensional shapes of proteins. The possibility that this may also hold for gene regulatory circuits has motivated an intense research effort to understand how the form or structure of such circuits (i.e., the wiring diagram of who regulates whom) governs their function (i.e., gene expression pattern)^6^.

Certain circuit structures — commonly referred to as *motifs*^7^ — are statistically enriched in the gene regulatory networks of organisms as diverse as bacteria^8^ and humans^9^. These structures include three-gene motifs such as the feedforward loop (TF *a* regulates TF *b*, and both regulate TF *c*)^10^ and four-gene motifs such as the bi-fan (TFs *a* and *b* both regulate TFs *c* and *d*)^11^. Such motifs are involved in important physiological and developmental processes, including response acceleration in the galactose utilization system of bacteria^12^ and the interpretation of morphogen gradients during embryogenesis in fruit flies^13^. The functions of these and other circuit motifs have therefore been the topic of several theoretical^10^,^13^,^14^,^15^,^16^,^17^ and experimental studies^12^,^18^,^19^.

A common interpretation of the statistical enrichment of a circuit motif is that it is a signature of adaptation, and that it reflects a function that the circuit performs. This view is contentious^20^,^21^,^22^,^23^ and several studies suggest that circuit function cannot always be inferred from circuit structure^11^,^24^,^25^,^26^, as structure, by itself, does not provide sufficient information to uniquely infer function. For example, the motif that drives circadian rhythms in *Drosophila melanogaster*^27^ can function either as a resonator or as an integrator, depending on various circuit parameters, such as protein degradation rates^25^. Despite such anecdotal examples, we still know very little about the extent to which a circuit’s function can be inferred from its form. This is because earlier studies of circuit function usually focused on just a few motifs and studied them only under a limited subset of all possible initial gene expression states. In addition, they only considered a few of the possible regulatory programs — the rules governing the dynamics of gene expression^28^,^29^,^30^ — that each motif implements. And furthermore, they did not consider the fact that regulatory circuits can be multifunctional^31^, forming distinct metastable gene expression patterns in different tissues^32^ and developmental stages^1^, or in response to different physiological conditions^33^. A systematic analysis of the relationship between form and function in gene regulatory circuits is therefore lacking.

To help fill this knowledge gap, we build upon our earlier work^31^,^34^ with Boolean circuits^35^ — a prominent model of genetic regulation — to systematically investigate the relationship between circuit form and function. We choose to study the Boolean model for three reasons. First, despite its many simplifying assumptions, the model and its variants have successfully recapitulated the gene expression patterns of regulatory circuits that drive processes as different as the embryonic specification of endomesoderm in the sea urchin^36^ and circadian oscillations in fungi and plants^37^. Moreover, the model has been widely adopted. Its application domains range from cell and developmental biology to community ecology^38^ and evolutionary robotics^39^, making the study of form and function in Boolean circuits broadly relevant. Second, the model allows one to study not only the structural variants of a circuit (i.e., motifs), but also the signal-integration logic of a circuit’s *cis*-regulatory regions. These regions specify a circuit’s regulatory program, encoding the input-output mapping of regulatory signals (e.g., the presence or absence of TFs) to gene expression patterns^28^,^29^,^30^. Third, small Boolean circuits are amenable to exhaustive enumeration; the maximum number of encodings of a circuit with *N* genes is 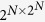, which remains a manageable number for small *N*. Importantly, this facilitates a comprehensive and systematic characterization of every regulatory program in every circuit motif under all possible initial gene expression states.

## Methods

**Figure 1:**
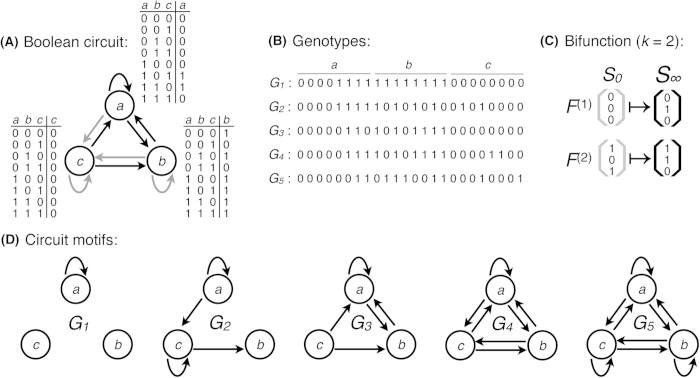
Schematic illustration of Boolean circuits. (**A**) A Boolean circuit with *N* = 3 genes, which are labeled as open circles *a*, *b*, *c*. Gene expression states are binary, such that genes are either on (1) or off (0). A directed edge *a* → *b* connects two genes if the expression state of *b* is dependent upon that of *a* (black arrows). Such dependencies are captured in the lookup table associated with each gene, which deterministically maps the 2^*N*^ possible expression states of *N* genes to an output expression state. These mappings may render some edges inactive (gray arrows). For example, the lookup table associated with gene *b* encodes the logical statement “*a* or not *c*”, which is independent of gene *b*. The autoregulatory interaction *b* → *b* is therefore inactive, as indicated by the gray arrow. (**B**) Five circuit genotypes *G*_*i*_, each represented as a vector of length *L* = *N* × 2^*N*^ = 24. Each genotype is constructed by concatenating the rightmost columns of the lookup tables of the circuit’s constituent genes. For example, the circuit shown in (**A**) is represented by the genotype *G*_3_, which — along with the circuits represented by the other four genotypes *G*_1_, *G*_2_, *G*_4_, *G*_5_ — has the bifunction shown in (**C**) i.e., 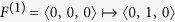, 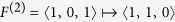. These circuits have other *k*-functions as well. For example, the circuit encoded by genotype *G*_3_ has a total of 12 distinct bifunctions, because six initial states result in the equilibrium state 〈0, 1, 0〉 and two initial states result in the equilibrium state 〈1, 1, 0〉. (**D**) The circuit motifs that correspond to the five genotypes in (**B**) illustrating how a circuit’s genotype determines its structure. Note how the inactive edges (gray arrows) in the circuit shown in (**A**) are not present in the motif that corresponds to genotype *G*_3_. The five motifs increase in complexity — measured as their number of edges^13^ — from left to right.

We consider Boolean circuits with *N* = 3 genes (Fig. 1A). Not only are circuits of this size the typical focus of circuit motif analyses^7^,^8^,^9^, they are also involved in important physiological and developmental processes. Examples include the *kaiABC* gene cluster in Cyanobacteria, which drives circadian oscillations^40^, and the *Oct4-Sall4-Nanog* circuit in mice, which controls pre-implantation development^41^. We compactly represent each circuit as a binary vector of length *L* = *N* × 2^*N*^ = 24, which we refer to as the circuit’s *genotype G* (Fig. 1B). The genotype specifies the signal-integration logic of the circuit’s constituent genes. In biological circuits, this logic is encoded by the number and affinity of TF binding sites in a gene’s *cis*-regulatory region^42^, in addition to the spacing between such sites^42^,^43^, their distance to the transcription start site^44^, and their genomic context^45^. The genotype *G* also specifies the circuit’s structure, since a circuit’s signal-integration logic may render some regulatory interactions inactive (gray arrows in Fig. 1A). Because different genotypes may encode the same regulatory interactions in different ways, a single motif may be represented by several genotypes. For example, in Fig. 1A, the autoregulatory interaction *b* → *b* is not present because the signal-integration logic of gene *b* encodes the statement “*a* or not *c*”. Different signal-integration logic may encode statements such as “*a* and *c*” or “not *a* and not *c*”, which are also independent of gene *b*, and would therefore render the autoregulatory interaction *b* → *b* inactive.

In the Boolean model, genes can be in one of two states, on (1) or off (0). Gene states are updated according to the states of the other genes in the circuit, as prescribed by the signal-integration logic encoded in the genotype. Specifically, for each gene *i*, the genotype encodes a mapping *f*_*i*_ that updates the gene’s state *σ*_*i*_(*t*) at time *t* according to
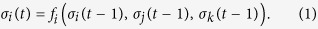
An example of such a mapping is shown as a look-up table in Fig. 1A, and all such mappings can be represented as a look-up table.

We refer to a circuit’s set of gene states at time *t* as a *gene expression state S*_*t*_. Each circuit is initialized with a gene expression state *S*_0_, which represents physiological conditions, such as the presence or absence of a sugar^12^ or hormone^46^, or exogenous regulatory influences, such as those from a higher level in a cell’s regulatory hierarchy^3^. From *S*_0_, the circuit’s gene expression state is updated deterministically and synchronously until it reaches an equilibrium expression state *S*_∞_ with period *p*, which can be a fixed-point (*p* = 1) or periodic (*p* > 1). Circuits with fixed-point expression patterns are exemplified by the gap gene circuit in *Drosophila melanogaster*, which interprets a maternally-deposited morphogen gradient to produce specific concentrations of protein along the anterior-posterior axis of the developing embryo. Examples of periodic expression patterns include circadian rhythms^47^ and the cell cycle^48^.

Each function that a circuit could potentially perform can be represented as a pairing of initial and equilibrium expression states, *F* = (*S*_0_, *S*_∞_)^31^,^34^. (Although a computational model can only be used to study potential circuit functions, we refer to them for brevity simply as functions.) This definition is motivated by circuits in development and physiology that produce specific, temporally invariant gene expression patterns in response to specific combinations of physiological or regulatory signals^1^. A circuit can have up to *k* ≤ 2^*N*^ functions. We refer to the set of these functions as a *multifunction* or as a *k-function*^31^,^34^: 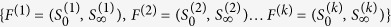. Multifunctional circuits are not unusual^32^,^33^,^49^,^50^,^51^ and are typified by the *hedgehog* circuit in butterflies, which both patterns the wing blade and helps to form the wing’s eyespots^52^. Our only requirements of a *k*-function are that the equilibrium expression states of its *k* constituent functions are all (i) fixed-points and (ii) unique (i.e., 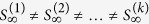). We use the terms *monofunction*, *bifunction*, *trifunction*, etc. to indicate the number of functions *k* in a multifunction. An example bifunction is shown in Fig. 1C.

We emphasize that a circuit can realize its *k* functions individually, or in various combinations, meaning that while a circuit genotype can be said to have between one and *k* functions, it can realize these functions in many different ways^31^. As an extreme example, a circuit with *k* = 8 functions (i.e., {*F*^(1)^ = (〈0, 0, 0〉, 〈0, 0, 0〉), *F*^(2)^ = (〈0, 0, 1〉, 〈0, 0, 1〉)…*F*^(8)^ = (〈1, 1, 1〉, 〈1, 1, 1〉)} can realize these functions in a total of 
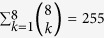
 distinct combinations. This is because a circuit’s functions vary depending on the initial expression states that the circuit experiences. Continuing with the example above, this circuit may either realize the bifunction {*F*^(1)^ = (〈0, 0, 0〉, 〈0, 0, 0〉), *F*^(2)^ = (〈0, 0, 1〉, 〈0, 0, 1〉)} or {*F*^(1)^ = (〈0, 0, 0〉, 〈0, 0, 0〉), *F*^(3)^ = (〈0, 1, 1〉, 〈0, 1, 1〉)}, and this distinction only depends upon the initial expression states experienced by the circuit.

The space of circuits we consider here comprises 2^*L*^ = 16, 777, 216 genotypes. For each of these genotypes, we determine the circuit’s motif (Fig. 1D) and all of its functions. This is accomplished by applying Eq. 1 to every genotype under all 2^*N*^ = 8 possible initial expression states *S*_0_ (i.e., physiological conditions or exogenous regulatory influences). Since there are only 32, 399 possible *k*-functions^31^,^34^ and 104 possible circuit motifs (after accounting for graph isomorphisms), both the genotype-to-function and the genotype-to-motif mappings are many-to-one. (We note that by including multiple arrow types — e.g., by explicitly distinguishing inhibitory interactions using blunted arrows^6^ — the number of circuit motifs would increase.) We explore these mappings to ask a series of questions about the form and function of Boolean circuits. These include (i) How many circuit motifs have more than one function? (ii) Are some of a motif’s functions realized by more circuit genotypes than others? (iii) How many distinct circuit motifs have the same function? (iv) How does multifunctionality constrain circuit structure?

## Results

### Most circuit genotypes have more than one function

**Figure 2:**
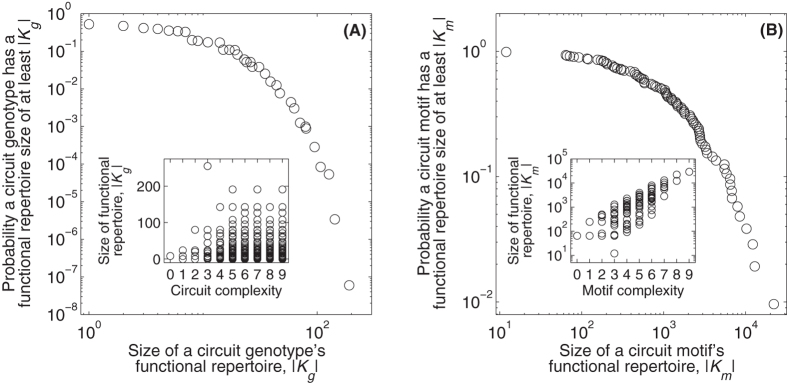
All circuit motifs have more than one *k*-function. Each data point shows the probability that a circuit (**A**) genotype or (**B**) motif has a functional repertoire size of at least |*K*_g_| or |*K*_m_|, respectively. The insets show the functional repertoire size in relation to the motif complexity, measured as the number of edges in the motif^13^, for (**A**) individual circuits and (**B**) all circuits with a given motif. Note the logarithmic scale of the x- and y-axes in the main panels and of the y-axis in the inset of (**B**).

To provide a baseline for comparison to the number of functions per circuit motif, we first determine the set *K*_g_ of functions per circuit genotype, which we refer to as the genotype’s *functional repertoire*. We denote the size of this set as |*K*_g_| and refer to a circuit as *viable* if it has at least one function (i.e., |*K*_g_| > 0). We reiterate that the maximum size of this set is 255, not 8, because a circuit can realize its functions individually or in various combinations^31^. Figure 2A shows the probability that a circuit genotype has a functional repertoire size of at least |*K*_g_|. Of the 16, 777, 216 possible genotypes, 11, 012, 415 (66%) have at least one function^31^. This implies that 34% of circuit genotypes are incapable of producing a fixed-point equilibrium expression pattern. Among the viable circuit genotypes, the median number of functions per circuit is seven; 74% of viable circuit genotypes have fewer than 10 functions and 92% have fewer than 25 functions. Only one circuit genotype realizes the maximum functional repertoire size of 255. It is the sole circuit genotype capable of an 8-function, a circuit with three edges: one autoregulatory interaction per gene. Finally, we observe no meaningful correlation between circuit complexity — measured as the number of active edges in the circuit — and the size of the circuit’s functional repertoire (Spearman’s *rho* = −0.008, *p* < 1 × 10^−50^; Fig. 2A, inset).

### All circuit motifs have more than one function

Previous studies have found that some circuit motifs have more than one function^11^,^24^,^25^,^26^. We explore the generality of this result by exhaustively analyzing the circuit functions of all 104 three-gene motifs, including all possible regulatory programs (i.e., signal-integration logic) and initial gene expression states. We refer to the set *K*_m_ of *k*-functions realized by a motif as the motif’s functional repertoire. We denote the size of this set as |*K*_m_|. Figure 2B shows the probability that a circuit motif has a functional repertoire size of at least |*K*_m_|. Every circuit motif has at least 12 *k*-functions and most circuit motifs realize many more. The motif with the fewest *k*-functions is the feedback loop; its functional repertoire of 12 *k*-functions includes 8 monofunctions and 4 bifunctions. The feedforward loop can realize 64 distinct *k*-functions, as can the motif with no regulatory interactions. In both cases, the functional repertoire comprises all of the 64 possible monofunctions. Remarkably, the median number of *k*-functions per circuit motif is 994 and the maximum number is 28, 990, which corresponds to the fully connected circuit. These observations suggest that motifs with more than one function are the rule, rather than the exception, in gene regulatory circuits.

We also find that the size of a motif’s functional repertoire is positively correlated with motif complexity (Spearman’s *r* = 0.78, *p* < 1.43 × 10^−22^; Fig. 2B, inset), despite the lack of correlation between the size of a circuit’s functional repertoire and its complexity (Fig. 2A, inset). Complex motifs, being rich in potential functionality, are therefore highly versatile.

### No one function dominates a motif’s functional repertoire

**Figure 3:**
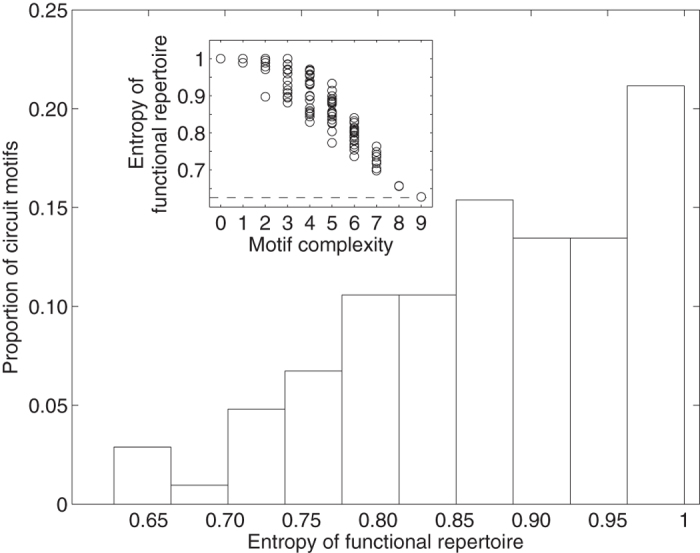
No one function dominates a motif’s functional repertoire. Entropy distribution of the functional repertoires of all 104 circuit motifs. The inset shows the entropy of a motif’s functional repertoire in relation to motif complexity. The dashed vertical line indicates the entropy of the functional repertoire of all 2^*L*^ circuits, regardless of their motif. This was calculated as 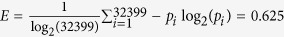, where *p*_*i*_ is the number of circuit genotypes with function *i* divided by 2^*L*^.

We have shown that each of the 104 three-gene motifs has more than one function. However, it may be the case that most of these functions are realized by just a small proportion of a motif’s constituent genotypes, while one or a few functions are realized by a large majority of these genotypes. This would indicate that a motif is much more likely to have some functions than others. To determine if this is the case, we calculate the entropy of a motif’s functional repertoire as 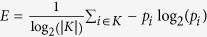, where *p*_*i*_ is the number *n*_*i*_ of a motif’s constituent genotypes that have function *i* divided by 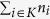. This measure takes on its maximum value of 1 when each of a motif’s functions are realized by an equal number of genotypes. It approaches its minimum value of 0 when a single function is realized by the vast majority of a motif’s genotypes. Figure 3 shows the distribution of this measure for all 104 circuit motifs. The distribution is skewed toward high entropy values, indicating that no one function dominates a motif’s functional repertoire.

We also find that the complexity of a motif and the entropy of its functional repertoire are inversely correlated (*r* = −0.89, *p* = 1.89 × 10^−36^; Fig. 3, inset). This indicates that while complex motifs are more functionally versatile than simple motifs (Fig. 2, inset), their constituent genotypes are not as uniformly distributed amongst these functions. We note that this observation is largely driven by a strong correlation between motif complexity and the number of circuit genotypes per motif (*r* = 0.90, *p* = 1.61 × 10^−38^). This is confirmed by a randomization procedure in which circuit genotypes are randomly assigned to circuit motifs while holding constant the number of genotypes per motif and the functional repertoire per genotype (see Supplementary Fig. S1 online). As the number of circuit genotypes per motif increases, the entropy of the functional repertoire approaches that of the entire space of 2^*L*^ circuit genotypes (Fig. 3, inset, dashed horizontal line), indicating that the low entropy of the functional repertoires of complex motifs is largely the result of a more complete sampling of the genotype space. However, even for the fully connected circuit, which has the lowest entropy (*E* = 0.627), the maximum proportion of circuit genotypes with any one function is only 0.02, and there are eight functions with this proportion of genotypes — all monofunctions in which the initial and equilibrium expression states are the same (e.g., *F* = (〈0, 0, 0〉, 〈0, 0, 0〉)). Thus, even for complex circuit motifs, no one function dominates the functional repertoire.

### Most functions are realized by more than one circuit motif

**Figure 4:**
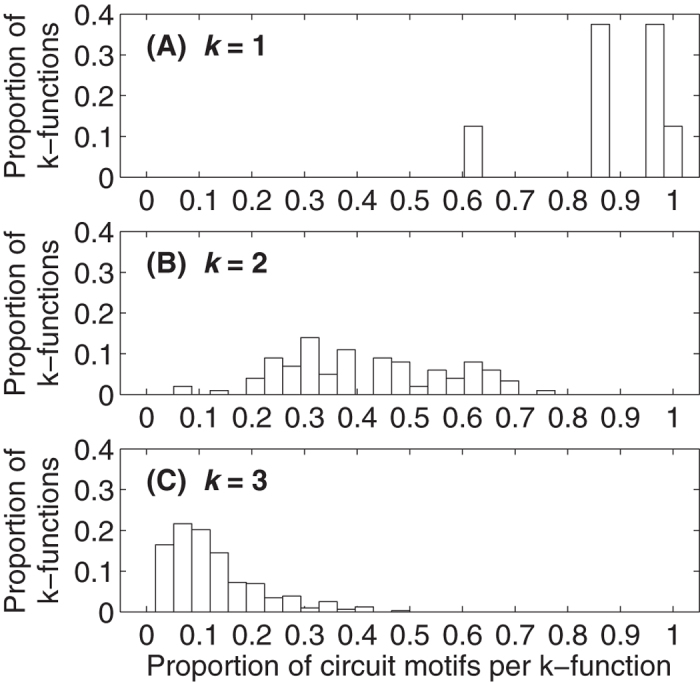
The same function can be realized by many distinct circuit motifs. The distribution of the proportion of all possible circuit motifs per *k*-function is shown for all (**A**) monofunctions, (**B**) bifunctions, and (**C**) trifunctions. Bar heights are therefore normalized by (**A**) 64, (**B**) 1204, and (**C**) 7616, the number of possible monofunctions, bifunctions, and trifunctions, respectively^31^.

Earlier theoretical^13^,^53^, empirical^54^,^55^, and experimental^19^ work has shown that certain circuit functions can be realized by more than one circuit motif. We explore the generality of this result by determining which of the 104 three-gene circuit motifs realize each *k*-function. Figure 4 shows the proportion of *k*-functions that are realized by a given proportion of the 104 circuit motifs, for all multifunctions with *k* ≤ 3. This figure indicates that circuit functions are generally realized by multiple circuit motifs. For example, of the 64 possible monofunctions^31^, all are realized by at least 60% of the 104 circuit motifs and 8 are realized by all 104 circuit motifs (Fig. 4A).

### Multifunctionality constrains circuit structure

**Figure 5:**
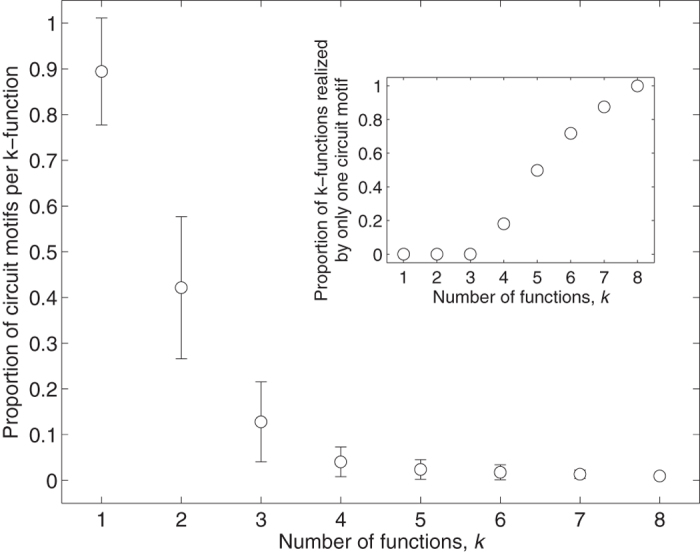
Multifunctionality constrains circuit structure. Data points show the average proportion of the 104 circuit motifs per *k*-function, in relation to the number of functions *k*. Error bars correspond to a single standard deviation. The inset shows the proportion of *k*-functions that are realized by just one circuit motif, in relation to the number of functions *k*.

In our previous work^31^,^56^, we found that the number of circuit genotypes that can have *k* functions decreases exponentially as the number of functions *k* increases, indicating that multifunctionality constrains the number of viable circuit genotypes. Figure 4 hints that multifunctionality may also constrain circuit structure, because the distributions shift toward the left as *k* increases. To assess the extent of this constraint, we consider all 32, 399 *k*-functions and quantify the proportion of the 104 circuit motifs that realize each of these *k*-functions. Figure 5 shows that this proportion decreases rapidly as the number of functions *k* increases, implying that multifunctionality severely constrains circuit structure. Said differently, the more functions a circuit needs to perform, the fewer circuit structures that can perform all of them. For example, the average monofunction is realized by 89% of circuit motifs, whereas the average bifunction is realized by only 42% of circuit motifs. This constraint is also evident in the proportion of *k*-functions that are realized by only a single circuit motif, which quickly approaches 1 as *k* exceeds 3 (Fig. 5, inset). In total, 23% of all *k*-functions (7353 of 32,399) are realized by only one circuit motif. While this clearly demonstrates that multifunctionality constrains circuit structure, the fact remains that 77% of all *k*-functions are realized by more than one circuit motif, indicating that it is generally not possible to directly infer circuit form from circuit function.

## Discussion

We have used a Boolean model of gene regulatory circuits^35^ to exhaustively characterize the gene expression patterns (functions) of nearly 17 million three-gene circuits and all possible structures (motifs) that these circuits can form, together with the functions they can perform, as embodied in the equilibrium gene expression patterns they reach from every possible initial gene expression state. Three main conclusions emerge. First, function does not follow form, that is, circuits with a given structure usually have multiple functions. Specifically, we found that every single one of the 104 possible three-gene motifs can perform at least 12 distinct functions and that nearly 90% of these motifs (93 of 104) can have more than one hundred functions. Moreover, each of a motif’s functions is realized by a roughly equal number of circuit genotypes, indicating that no one function dominates a motif’s functional repertoire. It is therefore not possible to directly infer circuit function from circuit structure without detailed information of the circuit’s signal-integration logic. These results complement and extend earlier observations that were based on the analysis of a small number of circuit motifs and on a limited subset of regulatory programs and initial gene expression states^11^,^24^,^25^,^26^. Further, they caution against the inference of circuit function from motif enrichment statistics^20^. However, it should be noted that the distribution of functions per motif depends crucially upon how the motifs are defined. By including inhibitory interactions, for example, the number of possible motifs would increase and some of these motifs may have fewer than the minimum number of 12 functions reported here (Fig. 2B).

Our second main conclusion is that form usually does not follow function, that is, circuits that can perform the same function can have very different structure. Over three-quarters of the 32,399 possible circuit functions are realized by more than one circuit motif. Remarkably, some functions are realized by all 104 motifs. These observations are in line with those from earlier computational models of large regulatory networks^57^,^58^, which found that many structurally distinct networks are capable of producing the same gene expression pattern. They are also in line with findings from comparative genomics, which have shown that the inter-species conservation of gene expression patterns does not imply conservation in the DNA sequences involved in regulating these expression patterns^59^,^60^,^61^,^62^. Such divergence in regulatory sequence has also lead to circuit rewiring^55^,^63^,^64^,^65^, as exemplified by the circuits controlling mating behavior in yeast^54^ and eye development in *Drosophila*^66^.

The third main conclusion is that multifunctionality constrains circuit structure. We found that the number of distinct circuit motifs with a given number of functions decreases rapidly as the number of functions increases. This observation may be relevant to synthetic biologists, who aim to understand the design constraints of circuits with specific biological functions. For example, a recent study designed several distinct three-gene circuit structures that are capable of interpreting a morphogen gradient to form a single spatial stripe^19^, inspired by the gene expression stripes formed during *Drosophila* embryogenesis. While such studies have understandably not yet considered multifunctional circuits, our results suggest that when they do, they will find far fewer circuit structures yielding multifunctions than monofunctions.

Our results also suggest that complex circuit motifs are more functionally versatile than simple motifs, as they have large functional repertoires that are not dominated by any one function. A recent analysis of the transcriptional regulatory networks of mouse and human revealed that complex motifs are statistically enriched, while simple motifs are statistically depleted, relative to randomized networks^61^. This suggests that in these organisms, highly versatile circuits are especially abundant. Combining our results with observations concerning the dynamical stability of circuit motifs^17^ may further our understanding of motif enrichment patterns in transcriptional regulatory networks.

Several studies have asked how functional constraints on transcriptional regulatory networks might favor some circuit motifs over others^58^,^67^,^68^,^69^. On the one hand, some studies have found that functional constraints lead to motif enrichment^67^,^69^. For instance, selection for oscillating gene expression patterns leads to the enrichment of certain four-gene motifs, such as the bifan^69^. On the other hand, a recent study focused on model regulatory networks capable of reproducing the gene expression patterns that embody flower organ specification in *Arabidopsis* showed that this functional constraint shapes the network’s pattern of edge usage (i.e., the presence or absence of specific regulatory interactions), but has almost no impact on motif enrichment^58^. Similar results were obtained in an earlier analysis of the set of regulatory networks capable of reproducing the gene expression pattern of the yeast cell cycle^68^.

Other studies have asked how the global topological properties of a network, such as its degree distribution, may influence local topological properties, such as the enrichment of motifs, even in the absence of functional constraints. For example, transcriptional regulatory networks are hierarchical and possess a heavy-tailed degree distribution, two global topological properties that have been shown to bias motif enrichment toward less complex motifs^70^. However, computational models have demonstrated that motif enrichment is easily fine-tuned via edge rewiring, even while global topological properties are held constant^71^, suggesting that it is possible, in principle, to select for particular motifs without changing the network’s global topological properties. Nevertheless, while selection for one of a motif’s several functions may lead to the enrichment of that motif, it remains impossible to use enrichment statistics to directly infer which of the motif’s several functions was selected. The reason is that, as we have shown, function does not follow form.

These findings highlight the limitations of diagrammatic representations of gene regulatory circuits, and underscore the importance of collecting detailed information about a circuit’s signal-integration logic^28^,^29^,^72^. Such information includes the location, number, orientation, order, and affinity of the regulating factors’ binding sites, whether the regulating factors interact cooperatively with themselves or with cofactors, and how target gene expression levels vary with the abundance and activity of the regulating factors^73^,^74^,^75^. We are only beginning to understand how these various facets of promoter architecture affect gene expression^42^,^43^,^44^,^76^, rendering the prediction of signal-integration logic from DNA sequence an outstanding challenge^77^.

In order to describe the circuit functions of all possible three-gene motifs under all possible regulatory programs and all possible initial gene expression states, we relied on a computational model of gene regulatory circuits. Specifically, we chose to study a Boolean model^35^ and to focus our attention on fixed-point gene expression patterns. These two decisions come with two caveats. First, the discrete nature of the model prohibits the analysis of protein production and degradation rates, which is a common theme in motif analyses^6^. We therefore cannot speak of a circuit as, for example, a “response accelerator”^12^ or as a “sign-sensitive delay”^14^. Second, our focus on fixed-point expression patterns necessarily excludes oscillating gene expression patterns, which are important for several biological functions, such as the cell cycle^48^. We were willing to accept these two caveats because they do not interfere with our ability to show that a circuit structure may have many functions, that a circuit function may be realized by many circuit structures, or that the number of circuit structures capable of driving multiple functions decreases with the number of functions. We therefore draw our three main conclusions: In gene regulatory circuits, function does not follow form, form rarely follows function, and form is severely constrained by multifunctionality.

## Additional Information

**How to cite this article**: Payne, J. L. and Wagner, A. Function does not follow form in gene regulatory circuits. *Sci. Rep.* **5**, 13015; doi: 10.1038/srep13015 (2015).

## Supplementary Material

Supplementary Information
