# Supplementary material for: Function does not follow form in gene regulatory circuits
Source: Sci Rep. 2015 Aug 20;5:13015. doi: 10.1038/srep13015 (PMC4542331; doi:10.1038/srep13015)
Supplement: Supplementary Information [file srep13015-s1.pdf]

Supplementary materials for:
Function does not follow form in gene regulatory circuits

Joshua L. Payne[1,2] & Andreas Wagner[1,2,3,*]

1. University of Zurich, Institute of Evolutionary Biology and Environmental Studies, Zurich, Switzerland

2. Swiss Institute of Bioinformatics, Lausanne, Switzerland

3. The Santa Fe Institute, Santa Fe, New Mexico, United States of America

*andreas.wagner@ieu.uzh.ch

Randomization tests for the entropy calculation

In the main text, we show that the entropy distribution of the functional repertoires of all 104 motifs is skewed toward high values (Fig. 3) and that the entropy of a motif's functional repertoire is inversely correlated with motif complexity (Fig. 3, inset). Here, we determine if these results can also be obtained by sampling circuit genotypes at random.

To do so, we randomly assign circuit genotypes to circuit motifs, while holding constant the number of circuit genotypes per motif and the functional repertoire of each circuit genotype. More specifically, for each motif i (that has n_i constituent genotypes), we randomly sample n_i circuit genotypes from the space of 2^L possible genotypes, with uniform probability and without replacement, and reassign these genotypes to motif i. We then calculate the entropy of motif i's functional repertoire, which is now made up of the functional repertoires of the randomly chosen circuit genotypes. We repeat this process 1000 times per circuit motif.

Figure S1A shows that when circuit genotypes are randomly assigned to circuit motifs, the entropy distribution does not exhibit the pronounced skew toward high values that was observed for the non-randomized data. This indicates that it is more likely for a small number of functions to dominate a functional repertoire in the randomized data than it is in the data analyzed in the main text.

Figure S1B shows that when circuit genotypes are randomly assigned to circuit motifs, the entropy of a motif's functional repertoire is inversely correlated with motif complexity ($r = -0.90, p = 1.95 \times 10^{-37}$). Moreover, as motif complexity increases, the entropy of a motif's functional repertoire approaches that of the entire space of 2^L circuit genotypes. Taken together with the positive correlation between motif

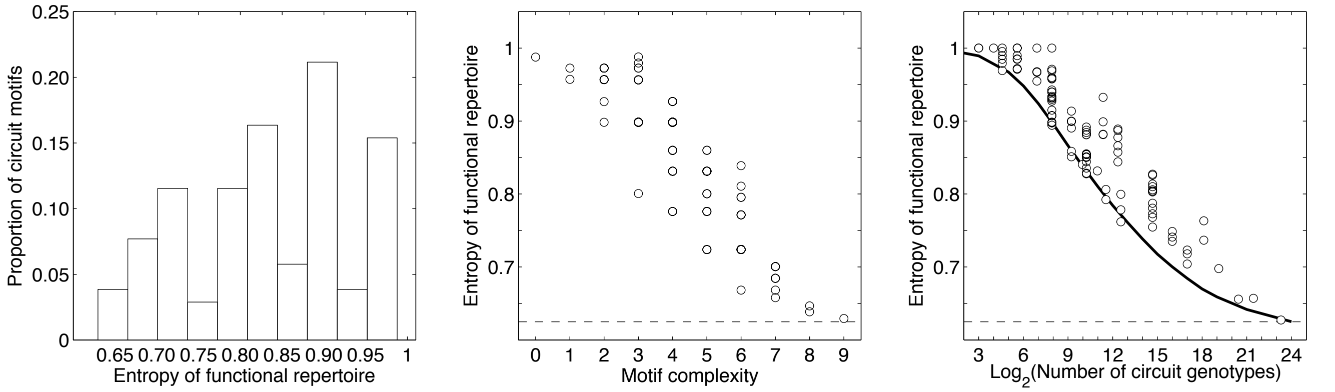

Figure S1. Entropy calculations for randomized data. (A) Entropy distribution of the functional repertoires of all 104 motifs, where the circuit genotypes have been randomly assigned to each motif, but the number of genotypes per motif and the functional repertoire of each circuit genotype have not been changed. (B) The entropy of a motif's functional repertoire in relation to motif complexity, using the same randomized data shown in (A). The dashed vertical line indicates the entropy of the functional repertoire of all 2^L circuits, regardless of their motif. (C) Entropy of the functional repertoire in relation to the logarithm of the number of circuit genotypes used to generate the repertoire. The solid line corresponds to randomized data, where circuit genotypes were sampled from the set of 2^L possible genotypes with uniform probability and without replacement. Open circles correspond to the non-randomized data shown in Fig. 3 of the main text. Dashed horizontal line is the same as in (B).

complexity and the number of circuit genotypes per motif ($r = 0.90, p = 1.61 \times 10^{-38}$), these results hint that motif entropy is driven primarily by the number of circuit genotypes per motif.

To determine if this is the case, we conduct a simpler sampling procedure. We choose x_i circuit genotypes at random, with uniform probability and without replacement, from the space of 2^L possible genotypes, where we systematically vary x_i from 2^2 to 2^{24} in 23 logarithmically spaced increments. As in the previous sampling procedure, the functional repertoire of each circuit genotype is left unchanged. We determine the entropy of the functional repertoire of the set of x_i circuit genotypes, and repeat this process 100 times for each x_i. The solid line in Figure S1C shows that the entropy of a functional repertoire of randomly chosen circuit genotypes decreases monotonically with the number of sampled genotypes, confirming that the number of circuit genotypes per motif is the main driver of the relationship between entropy and motif complexity (Fig. 3, inset). However, by plotting the entropy of the functional repertoires of the 104 motifs in relation to their number of constituent genotypes (open circles in Fig. S1C), it is evident that entropy is generally higher in the functional repertoires of the 104 motifs than in the functional repertoires of the same number of randomly selected circuit genotypes.
